# Supplementary material for: Construction of a High-Density Genetic Map and Mapping of Firmness in Grapes (Vitis vinifera L.) Based on Whole-Genome Resequencing
Source: Int J Mol Sci. 2020 Jan 25;21(3):797. doi: 10.3390/ijms21030797 (PMC7037167; doi:10.3390/ijms21030797)
Supplement: Supplementary file 1 [file ijms-21-00797-s001.pdf]

Table S1. Summary and mapping statistics of genome re-sequencing data of hybrids and parents.

Sample	Length (bp)	Clean Reads	Clean Data (bp)	Q30 (%)	GC Rate (%)	Coverage Rate (%)	Map Bases Rate (%)	Uni Hit Bases Rate(%)	Effective Depth (×)
Mascat Hamburg ♀	150	145657038	21848555700	92.88	37.24	94.26	96.3	71.76	44.93
Crimson Seedless ♂	150	152526478	22878971700	92.66	37.51	93.49	95.48	70.39	47.05
J1	150	54356636	8153495400	93.06	36.53	92.95	96.34	68.09	16.77
J2	150	54073294	8110994100	93.28	36.57	91.61	96.43	69.36	16.68
J3	150	48914452	7337167800	93.30	36.63	91.34	96.64	68.93	15.09
J4	150	49289762	7393464300	93.21	36.49	91.92	96.27	71.83	15.20
J5	150	56565216	8484782400	93.16	36.20	92.22	96.32	68.60	17.45
J6	150	37840098	5676014700	93.51	36.60	91.61	96.45	79.14	11.67
J7	150	57478814	8621822100	93.31	36.56	92.09	96.71	69.04	17.73
J9	150	42282434	6342365100	93.31	36.28	90.95	96.84	69.88	13.04
J10	150	48116080	7217412000	92.93	36.45	91.89	96.47	70.32	14.84
J11	150	44665020	6699753000	93.12	36.39	91.79	96.34	70.58	13.78
J12	150	56764950	8514742500	93.41	36.33	92.27	96.46	67.96	17.51
J13	150	44460794	6669119100	93.17	36.45	91.4	96.01	69.84	13.71
J14	150	48207946	7231191900	93.51	36.48	92.18	96.74	78.35	14.87
J15	150	53393660	8009049000	93.39	36.78	92.30	96.46	70.78	16.47
J16	150	53974290	8096143500	93.32	36.55	92.05	95.47	69.04	16.65
J17	150	47105986	7065897900	93.53	36.86	91.33	96.03	71.07	14.53
J19	150	57169648	8575447200	93.23	36.62	91.81	96.38	70.37	17.64
J20	150	59497052	8924557800	93.50	36.46	92.03	96.45	69.19	18.35
J24	150	52311360	7846704000	93.24	36.63	91.83	95.10	67.44	16.14
J25	150	44072876	6610931400	93.63	36.56	91.14	96.48	68.71	13.60
J27	150	49347012	7402051800	93.43	36.69	91.86	96.44	68.45	15.22
J28	150	48602326	7290348900	92.97	36.81	92.09	96.32	68.47	14.99
J29	150	47194466	7079169900	93.00	36.77	91.56	96.05	68.36	14.56
J30	150	54402750	8160412500	92.60	36.92	92.09	96.40	69.93	16.78
J31	150	52619468	7892920200	93.50	36.79	92.04	96.26	67.75	16.23
J33	150	64295506	9644325900	93.13	36.94	92.20	96.58	67.74	19.83

J35	150	55064188	8259628200	93.19	36.6	91.35	96.15	68.38	16.99
J36	150	58654068	8798110200	93.16	36.94	91.48	96.33	68.61	18.09
J37	150	46181194	6927179100	93.53	36.75	91.47	96.65	69.38	14.25
J39	150	50773260	7615989000	93.39	36.52	90.91	96.31	68.19	15.66
J40	150	46212874	6931931100	93.22	36.77	91.83	96.58	69.06	14.26
J41	150	47547372	7132105800	93.03	37.05	91.45	96.08	68.48	14.67
J42	150	48946796	7342019400	93.35	36.91	91.65	96.36	70.50	15.10
J43	150	44048252	6607237800	93.49	36.54	91.67	96.11	76.25	13.59
J44	150	42302214	6345332100	92.97	36.75	91.13	96.44	70.48	13.05
J45	150	53047626	7957143900	93.59	36.75	91.64	96.10	69.01	16.36
J46	150	51659954	7748993100	91.96	36.85	91.85	96.01	67.36	15.94
J47	150	57732310	8659846500	93.22	36.43	92.43	96.5	67.66	17.81
J49	150	53975532	8096329800	93.25	36.26	91.49	96.23	68.54	16.65
J50	150	58290834	8743625100	93.15	36.69	91.87	96.36	68.30	17.98
J51	150	55955780	8393367000	93.23	36.63	91.49	96.31	67.14	17.26
J52	150	64930284	9739542600	92.94	36.84	92.50	96.30	68.57	20.03
J53	150	55642704	8346405600	92.91	36.85	92.42	96.21	69.03	17.16
J54	150	42368148	6355222200	93.57	36.40	91.35	96.40	77.10	13.07
J55	150	46890824	7033623600	93.38	36.60	91.43	96.36	71.67	14.46
J56	150	53114834	7967225100	93.13	36.72	91.76	96.43	67.35	16.38
J57	150	55673878	8351081700	93.44	36.37	91.84	96.75	67.02	17.17
J58	150	55472644	8320896600	93.18	36.27	91.82	96.50	71.02	17.11
J60	150	50029196	7504379400	92.71	37.07	91.87	96.31	70.87	15.43
J61	150	56875828	8531374200	92.91	36.60	91.92	96.4	69.29	17.54
J62	150	52981368	7947205200	92.64	36.92	91.57	95.9	71.25	16.34
J63	150	49934136	7490120400	92.55	36.99	91.92	96.44	70.57	15.40
J64	150	57680396	8652059400	92.82	37.07	91.61	96.26	68.9	17.79
J65	150	49223354	7383503100	92.75	37.18	91.51	95.99	69.61	15.18
J67	150	42966780	6445017000	93.00	36.64	91.35	96.12	70.92	13.25
J68	150	44983640	6747546000	92.42	37.28	91.04	95.79	70.9	13.88
J69	150	42163032	6324454800	93.23	36.68	91.56	96.52	78.83	13.01
J70	150	47117258	7067588700	92.81	36.73	91.41	96.46	71.26	14.53
J72	150	49247832	7387174800	93.35	36.61	91.89	96.31	76.64	15.19
J75	150	43328822	6499323300	92.52	36.77	91.50	96.14	71.45	13.37

J76	150	49026984	7354047600	92.80	37.29	91.72	96.07	69.51	15.12
J77	150	53217240	7982586000	93.04	36.99	91.5	96.39	68.12	16.42
J78	150	64985714	9747857100	93.33	36.95	92.24	96.58	68.03	20.05
J80	150	57630602	8644590300	92.61	37.18	92.68	96.18	70.44	17.78
J81	150	45872888	6880933200	93.20	36.94	91.37	96.11	77.32	14.15
J83	150	44099540	6614931000	93.45	36.72	91.55	96.00	72.70	13.60
J85	150	44489636	6673445400	92.95	37.86	91.35	96.04	70.75	13.72
J86	150	46302318	6945347700	93.82	36.63	91.66	96.53	71.72	14.28
J87	150	65535548	9830332200	93.37	36.60	91.97	96.61	70.90	20.22
J89	150	55707088	8356063200	93.78	36.50	91.53	96.28	70.02	17.18
J90	150	53554676	8033201400	93.90	36.91	92.54	96.31	70.86	16.52
J91	150	49427918	7414187700	93.81	37.60	91.10	96.14	70.62	15.25
J92	150	56970024	8545503600	94.05	36.41	91.87	96.53	69.79	17.57
J93	150	48400486	7260072900	94.05	37.21	91.07	96.36	71.46	14.93
J94	150	48251552	7237732800	93.64	37.01	91.77	96.59	70.06	14.88
J96	150	54162762	8124414300	93.63	36.63	91.96	96.49	69.98	16.71
J97	150	46565968	6984895200	93.71	37.00	91.02	95.91	70.89	14.36
J98	150	46279870	6941980500	93.33	36.78	91.48	96.61	71.48	14.28
J99	150	59625026	8943753900	93.42	36.42	91.74	96.52	70.82	18.39
J101	150	45876390	6881458500	93.67	36.76	90.98	96.18	72.17	14.15
J102	150	46875548	7031332200	93.31	36.75	91.44	96.48	73.21	14.46
J106	150	52248418	7837262700	93.56	36.62	91.78	96.22	71.63	16.12
J107	150	50148798	7522319700	93.21	36.54	91.63	96.52	71.27	15.47
J108	150	44166836	6625025400	93.50	36.71	91.63	96.5	77.89	13.62
J110	150	55238878	8285831700	92.92	37.41	91.71	96.48	69.23	17.04
J111	150	50169044	7525356600	93.03	36.56	91.59	96.20	69.6	15.48
J113	150	47377364	7106604600	92.90	36.76	91.44	96.32	70.73	14.61
J114	150	45190664	6778599600	93.45	36.52	91.86	96.33	72.00	13.94
J115	150	49067250	7360087500	93.22	36.72	91.08	96.32	71.32	15.14
J116	150	65234912	9785236800	93.50	36.63	92.06	96.41	70.80	20.12
J117	150	52810780	7921617000	93.19	37.38	91.27	96.33	71.07	16.29
J118	150	52315968	7847395200	92.83	37.29	91.95	96.39	71.49	16.14
J119	150	54619830	8192974500	92.57	37.10	91.84	96.20	71.54	16.85
J120	150	53327244	7999086600	92.59	36.95	92.59	95.96	72.60	16.45

J121	150	58106240	8715936000	92.76	36.85	92.67	96.46	70.05	17.92
J122	150	45213372	6782005800	92.53	37.71	91.45	96.27	72.10	13.95
J123	150	44999150	6749872500	93.045	37.74	91.56	96.50	73.99	13.88
J124	150	47407802	7111170300	93.30	37.18	91.54	95.53	76.59	14.62
J125	150	47113670	7067050500	93.17	37.67	90.86	96.23	72.94	14.53
J126	150	40789428	6118414200	93.10	37.11	91.54	96.63	75.66	12.58
J127	150	49057054	7358558100	93.37	37.02	91.86	96.33	78.48	15.13
J128	150	52834030	7925104500	93.22	37.23	91.89	96.54	72.97	16.30
J129	150	55661838	8349275700	93.71	36.70	91.20	96.45	72.46	17.17
J130	150	52352204	7852830600	93.03	38.37	91.01	95.99	71.43	16.15
J131	150	55045800	8256870000	93.46	36.64	92.13	96.54	72.64	16.98

Table S2. Characteristics of the linkage groups of Muscat hamburg genetic map.

Linkage group	Chromosomes	Length (cM)	Number of SNP Markers	Number of Bin Markers	Max Gap (cM)	Gap <5cM (%)	Mean Markers Distance (cM)
LG1	1	54.07	298	29	10.74	99.33	1.86
LG2	2	70.38	226	37	7.75	98.67	1.90
LG3	3	77.35	42	20	46.52	95.24	3.87
LG4	4	74.15	625	49	5.80	99.68	1.51
LG5	5	102.46	523	61	14.85	99.62	1.68
LG6	6	16.36	53	14	3.85	100.00	1.17
LG7	7	98.14	650	72	3.85	100.00	1.36
LG8	8	79.93	395	53	6.77	99.24	1.51
LG9	9	76.15	1425	57	10.74	99.93	1.34
LG10	10	98.41	1440	50	21.41	99.93	1.97
LG11	11	66.87	384	38	13.80	99.48	1.76
LG12	12	95.89	458	59	16.97	99.56	1.63
LG13	13	100.75	983	38	27.47	99.49	2.65
LG14	14	94.89	1115	62	16.97	99.91	1.53
LG15	15	73.16	920	44	4.82	100.00	1.66
LG16	16	99.07	930	86	3.85	100.00	1.15
LG17	17	31.79	177	21	5.80	98.87	1.51
LG18	18	97.21	1344	71	6.77	99.85	1.37
LG19	19	87.53	961	74	2.89	100.00	1.18
Total		1494.56	12949	935			
Average		78.66	682	49.21	12.19	99.41	1.60

Table S3. Characteristics of the linkage groups of Crimson seedless genetic map.

Linkage group	Chromosomes	Length (cM)	Number of SNP Markers	Number of Bin Markers	Max Gap (cM)	Gap <5cM (%)	Mean Markers Distance (cM)
LG1	1	76.20	105	38	7.75	96.19	2.01
LG2	2	68.31	325	49	3.85	100.00	1.39
LG3	3	105.17	436	47	35.88	99.77	2.24
LG4	4	87.84	108	39	32.89	97.22	2.25
LG5	5	90.40	798	36	20.27	99.62	2.51
LG6	6	79.91	254	47	5.80	99.61	1.70
LG7	7	85.77	525	55	9.74	99.62	1.56
LG8	8	60.76	77	30	7.75	94.81	2.03
LG9	9	79.95	1299	53	7.75	99.85	1.51
LG10	10	77.07	1081	51	8.74	99.82	1.51
LG11	11	56.83	394	42	8.74	99.75	1.35
LG12	12	82.74	886	67	4.82	100.00	1.23
LG13	13	91.51	1099	59	7.75	99.82	1.55
LG14	14	68.49	432	39	9.74	99.54	1.76
LG15	15	92.85	985	49	13.80	99.70	1.89
LG16	16	105.90	1730	76	20.27	99.88	1.39
LG17	17	-	-		-	-	-
LG18	18	102.20	2178	71	11.75	99.95	1.44
LG19	19	82.78	545	65	7.75	99.82	1.27
Total		1494.67	13257	913			
Average		83.04	737	50.72	12.50	99.17	1.64

Table S4. List of total genes for berry firmness identified in the quantitative trait loci region located on linkage group 18.

Gene ID	Function Description
VIT_18s0041g00250	PREDICTED: TMV resistance protein N-like
VIT_18s0041g00260	hypothetical protein VITISV_006408
VIT_18s0041g00270	hypothetical protein VITISV_006408
VIT_18s0041g00280	PREDICTED: uncharacterized protein LOC100252816 isoform X1
VIT_18s0041g00290	PREDICTED: E3 ubiquitin-protein ligase RNF170
VIT_18s0041g00330	PREDICTED: uncharacterized protein LOC100244294 isoform X2
VIT_18s0041g00340	PREDICTED: receptor-like protein 12
VIT_18s0041g00350	PREDICTED: carboxymethylenebutenolidase homolog
VIT_18s0041g00360	PREDICTED: uncharacterized protein LOC100261433
VIT_18s0041g00370	PREDICTED: cysteine proteinase inhibitor 8-like
VIT_18s0041g00380	PREDICTED: DNA repair protein XRCC4 isoform X3
VIT_18s0041g00390	PREDICTED: cysteine proteinase inhibitor 8-like
VIT_18s0041g00400	unnamed protein product
VIT_18s0041g00410	unnamed protein product
VIT_18s0041g00420	hypothetical protein VITISV_013162
VIT_18s0041g00430	PREDICTED: patellin-3
VIT_18s0041g00440	unnamed protein product
VIT_18s0041g00450	unnamed protein product
VIT_18s0041g00480	PREDICTED: uncharacterized protein LOC104878615, partial
VIT_18s0041g00490	hypothetical protein VITISV_000973
VIT_18s0041g00500	unnamed protein product
VIT_18s0041g00510	unnamed protein product
VIT_18s0041g00520	PREDICTED: protein NRT1/ PTR FAMILY 5.10-like isoform X2
VIT_18s0041g00530	unnamed protein product
VIT_18s0041g00540	unnamed protein product
VIT_18s0041g00550	PREDICTED: protein NRT1/ PTR FAMILY 5.10-like
VIT_18s0041g00560	PREDICTED: protein NRT1/ PTR FAMILY 5.10-like
VIT_18s0041g00570	unnamed protein product
VIT_18s0041g00580	PREDICTED: uncharacterized protein LOC100854586
VIT_18s0041g00590	PREDICTED: protein NRT1/ PTR FAMILY 5.10-like
VIT_18s0041g00600	PREDICTED: protein NRT1/ PTR FAMILY 5.10-like
VIT_18s0041g00610	unnamed protein product

VIT_18s0041g00630	PREDICTED: protein NRT1/ PTR FAMILY 5.10-like
VIT_18s0041g00640	unnamed protein product
VIT_18s0041g00650	PREDICTED: protein NRT1/ PTR FAMILY 5.10-like
VIT_18s0041g00660	PREDICTED: protein NRT1/ PTR FAMILY 5.10-like
VIT_18s0041g00670	PREDICTED: protein NRT1/ PTR FAMILY 5.10
VIT_18s0041g00680	PREDICTED: transmembrane 9 superfamily member 1
VIT_18s0041g00690	PREDICTED: transcription factor bHLH80-like isoform X2
VIT_18s0041g00700	**PREDICTED: NAC domain-containing protein 90-like**
VIT_18s0041g00710	PREDICTED: anthocyanidin 5,3-O-glucosyltransferase
VIT_18s0041g00720	unnamed protein product
VIT_18s0041g00730	hypothetical protein VITISV_020937
VIT_18s0041g00740	hypothetical protein VITISV_020938
VIT_18s0041g00750	unnamed protein product
VIT_18s0041g00770	unnamed protein product
VIT_18s0041g00790	PREDICTED: anthocyanidin 5,3-O-glucosyltransferase
VIT_18s0041g00800	PREDICTED: anthocyanidin 5,3-O-glucosyltransferase-like
VIT_18s0041g00810	PREDICTED: UDP-glycosyltransferase 88A1-like
VIT_18s0041g00830	hypothetical protein VITISV_020131
VIT_18s0041g00840	PREDICTED: UDP-glycosyltransferase 88A1-like
VIT_18s0041g00850	unnamed protein product
VIT_18s0041g00860	unnamed protein product
VIT_18s0041g00870	unnamed protein product
VIT_18s0041g00890	hypothetical protein VITISV_029184
VIT_18s0041g00900	PREDICTED: anthocyanidin 5,3-O-glucosyltransferase-like
VIT_18s0041g00910	PREDICTED: UDP-glycosyltransferase 88A1-like
VIT_18s0041g00920	PREDICTED: UDP-glycosyltransferase 88A1-like
VIT_18s0041g00930	PREDICTED: anthocyanidin 5,3-O-glucosyltransferase-like, partial
VIT_18s0041g00940	unnamed protein product
VIT_18s0041g00950	PREDICTED: UDP-glycosyltransferase 88A1-like
VIT_18s0041g00960	unnamed protein product
VIT_18s0041g00970	PREDICTED: anthocyanidin 5,3-O-glucosyltransferase
VIT_18s0041g00980	PREDICTED: UDP-glycosyltransferase 88A1-like
VIT_18s0041g01000	PREDICTED: UDP-glycosyltransferase 88A1-like
VIT_18s0041g01010	hypothetical protein VITISV_001706

VIT_18s0041g01030	unnamed protein product
VIT_18s0041g01040	PREDICTED: UDP-glycosyltransferase 88A1-like
VIT_18s0041g01050	unnamed protein product
VIT_18s0041g01080	PREDICTED: UDP-glycosyltransferase 88A1
VIT_18s0041g01090	PREDICTED: F-box/kelch-repeat protein At3g23880-like
VIT_18s0041g01100	unnamed protein product
VIT_18s0041g01110	PREDICTED: pentatricopeptide repeat-containing protein At3g13880 isoform X1
VIT_18s0041g01130	hypothetical protein VITISV_028402
VIT_18s0041g01140	PREDICTED: probable L-type lectin-domain containing receptor kinase S.5
VIT_18s0041g01150	PREDICTED: uncharacterized protein LOC100246555
VIT_18s0041g01160	PREDICTED: probable L-type lectin-domain containing receptor kinase S.5
VIT_18s0041g01170	PREDICTED: probable L-type lectin-domain containing receptor kinase S.5
VIT_18s0041g01180	unnamed protein product
VIT_18s0041g01190	unnamed protein product
VIT_18s0041g01210	PREDICTED: probable L-type lectin-domain containing receptor kinase S.5 isoform X2
VIT_18s0041g01220	hypothetical protein VITISV_006706
VIT_18s0041g01230	PREDICTED: stromal 70 kDa heat shock-related protein, chloroplastic-like
VIT_18s0041g01240	PREDICTED: amino acid permease 2-like
VIT_18s0041g01250	PREDICTED: amino acid permease 8-like
VIT_18s0041g01260	PREDICTED: type 1 phosphatases regulator ypi1-like
VIT_18s0041g01270	unnamed protein product
VIT_18s0041g01280	unnamed protein product
VIT_18s0041g01290	PREDICTED: serine/threonine-protein kinase-like protein CCR4
VIT_18s0041g01330	unnamed protein product
VIT_18s0041g01340	PREDICTED: TMV resistance protein N-like
VIT_18s0041g01350	PREDICTED: receptor-like protein kinase HAIKU2
VIT_18s0041g01360	PREDICTED: protein TIC 40, chloroplastic isoform X1
VIT_18s0041g01370	unnamed protein product
VIT_18s0041g01380	hypothetical protein VITISV_024045
VIT_18s0041g01390	unnamed protein product
VIT_18s0041g01400	PREDICTED: TMV resistance protein N-like
VIT_18s0041g01410	hypothetical protein VITISV_024045
VIT_18s0041g01420	unnamed protein product
VIT_18s0041g01430	unnamed protein product

VIT_18s0041g01440	hypothetical protein VITISV_013693
VIT_18s0041g01450	PREDICTED: uncharacterized protein LOC100266406
VIT_18s0041g01460	hypothetical protein VITISV_037610
VIT_18s0041g01470	PREDICTED: TMV resistance protein N-like
VIT_18s0041g01500	unnamed protein product
VIT_18s0041g01510	hypothetical protein VITISV_013692
VIT_18s0041g01520	PREDICTED: TMV resistance protein N-like
VIT_18s0041g01530	PREDICTED: beta-amyrin 28-oxidase-like
VIT_18s0041g01550	uncharacterized protein LOC102577679 [Solanum tuberosum]
VIT_18s0041g01560	unnamed protein product
VIT_18s0041g01570	PREDICTED: TMV resistance protein N-like
VIT_18s0041g01580	PREDICTED: beta-amyrin 28-oxidase
VIT_18s0041g01590	PREDICTED: beta-amyrin 28-oxidase
VIT_18s0041g01600	unnamed protein product
VIT_18s0041g01610	PREDICTED: uncharacterized protein LOC104882743 isoform X2
VIT_18s0041g01620	PREDICTED: TMV resistance protein N-like isoform X2
VIT_18s0041g01630	unnamed protein product
VIT_18s0041g01640	unnamed protein product
VIT_18s0041g01650	PREDICTED: TMV resistance protein N-like, partial
VIT_18s0041g01680	unnamed protein product
VIT_18s0041g01700	unnamed protein product
VIT_18s0041g01710	PREDICTED: TMV resistance protein N-like, partial
VIT_18s0041g01720	unnamed protein product
VIT_18s0041g01730	unnamed protein product
VIT_18s0041g01740	unnamed protein product
VIT_18s0041g01750	PREDICTED: TMV resistance protein N-like
VIT_18s0041g01770	unnamed protein product
VIT_18s0041g01780	PREDICTED: cyprosin-like isoform X1
VIT_18s0041g01790	PREDICTED: TMV resistance protein N-like
VIT_18s0041g01800	PREDICTED: beta-amyrin 28-oxidase
VIT_18s0041g01810	unnamed protein product
VIT_18s0041g01820	unnamed protein product
VIT_18s0041g01830	PREDICTED: glyoxylate/hydroxypyruvate reductase A HPR2 isoform X3
VIT_18s0041g01840	PREDICTED: uncharacterized protein LOC100263649

VIT_18s0041g01860	unnamed protein product
VIT_18s0041g01870	unnamed protein product
VIT_18s0041g01890	PREDICTED: uncharacterized protein LOC100854818
VIT_18s0041g01900	PREDICTED: elongation factor 1-gamma
VIT_18s0041g01920	unnamed protein product
VIT_18s0041g01930	PREDICTED: pentatricopeptide repeat-containing protein At4g14050, mitochondrial-like
VIT_18s0041g01940	PREDICTED: uncharacterized protein LOC100260177 isoform X1
VIT_18s0041g01980	unnamed protein product
VIT_18s0041g02010	PREDICTED: putative 12-oxophytodienoate reductase 11
VIT_18s0041g02020	PREDICTED: putative 12-oxophytodienoate reductase 11
VIT_18s0041g02040	PREDICTED: putative 12-oxophytodienoate reductase 11
VIT_18s0041g02050	PREDICTED: putative 12-oxophytodienoate reductase 11
VIT_18s0041g02060	PREDICTED: putative 12-oxophytodienoate reductase 11
VIT_18s0041g02070	PREDICTED: putative 12-oxophytodienoate reductase 11
VIT_18s0041g02080	PREDICTED: putative 12-oxophytodienoate reductase 11
VIT_18s0041g02090	unnamed protein product
VIT_18s0041g02100	unnamed protein product
VIT_18s0041g02110	PREDICTED: uncharacterized protein LOC100265490 isoform X1
VIT_18s0041g02120	PREDICTED: TMV resistance protein N-like
VIT_18s0041g02130	unnamed protein product
VIT_18s0041g02140	**PREDICTED: agamous-like MADS-box protein AGL12**
VIT_18s0041g02150	unnamed protein product
VIT_18s0041g02160	PREDICTED: GDSL esterase/lipase At1g33811
VIT_18s0041g02170	PREDICTED: uncharacterized protein LOC100241456 isoform X1
VIT_18s0041g02180	PREDICTED: uncharacterized protein LOC100246577
VIT_18s0041g02190	PREDICTED: protein DA1-related 4-like
VIT_18s0041g02200	unnamed protein product
VIT_18s0041g02210	PREDICTED: TMV resistance protein N-like
VIT_18s0041g02220	PREDICTED: uncharacterized protein LOC100249874
VIT_18s0041g02230	unnamed protein product
VIT_18s0041g02240	unnamed protein product
VIT_18s0041g02250	unnamed protein product
VIT_18s0041g02260	hypothetical protein VITISV_011309

VIT_18s0041g02270	unnamed protein product
VIT_18s0041g02290	unnamed protein product
VIT_18s0041g02300	unnamed protein product
VIT_18s0041g02310	unnamed protein product
VIT_18s0041g02320	unnamed protein product
VIT_18s0041g02330	unnamed protein product
VIT_18s0041g02350	unnamed protein product
VIT_18s0041g02360	unnamed protein product
VIT_18s0041g02370	unnamed protein product
VIT_18s0041g02380	unnamed protein product
VIT_18s0041g02390	unnamed protein product
VIT_18s0041g02400	unnamed protein product
VIT_18s0041g02410	**PREDICTED: abscisic-aldehyde oxidase-like**
VIT_18s0041g02440	PREDICTED: TMV resistance protein N-like
VIT_18s0041g02450	unnamed protein product
VIT_18s0041g02470	PREDICTED: putative disease resistance protein At4g11170, partial
VIT_18s0089g00010	unnamed protein product
VIT_18s0089g00020	unnamed protein product
VIT_18s0089g00030	PREDICTED: SCY1-like protein 2
VIT_18s0089g00040	PREDICTED: uncharacterized protein LOC100261885
VIT_18s0089g00050	PREDICTED: uncharacterized protein LOC100261885
VIT_18s0089g00060	hypothetical protein VITISV_032106
VIT_18s0089g00070	unnamed protein product
VIT_18s0089g00080	unnamed protein product
VIT_18s0089g00090	unnamed protein product
VIT_18s0089g00100	unnamed protein product
VIT_18s0089g00110	PREDICTED: uncharacterized protein LOC104877471
VIT_18s0089g00120	unnamed protein product
VIT_18s0089g00140	unnamed protein product
VIT_18s0089g00160	PREDICTED: mannan endo-1,4-beta-mannosidase 5
VIT_18s0089g00170	PREDICTED: mannan endo-1,4-beta-mannosidase 5
VIT_18s0089g00180	unnamed protein product
VIT_18s0089g00190	PREDICTED: mannan endo-1,4-beta-mannosidase 5
VIT_18s0089g00200	PREDICTED: mannan endo-1,4-beta-mannosidase 5

VIT_18s0089g00210	**PREDICTED: endoglucanase 3**
VIT_18s0089g00220	PREDICTED: splicing factor 3B subunit 4
VIT_18s0089g00250	unnamed protein product
VIT_18s0089g00260	PREDICTED: pentatricopeptide repeat-containing protein At1g71420-like
VIT_18s0089g00270	PREDICTED: TMV resistance protein N-like
VIT_18s0089g00280	hypothetical protein VITISV_026446
VIT_18s0089g00290	PREDICTED: pentatricopeptide repeat-containing protein At1g71420-like
VIT_18s0089g00300	unnamed protein product
VIT_18s0089g00310	PREDICTED: uncharacterized protein LOC100854344
VIT_18s0089g00340	PREDICTED: uncharacterized protein slp1
VIT_18s0089g00350	PREDICTED: dynamin-2A
VIT_18s0089g00360	PREDICTED: eukaryotic translation initiation factor 2D
VIT_18s0089g00370	PREDICTED: probable carboxylesterase 18
VIT_18s0089g00380	PREDICTED: probable carboxylesterase 18
VIT_18s0089g00390	PREDICTED: probable carboxylesterase 18
VIT_18s0089g00400	PREDICTED: probable carboxylesterase 18
VIT_18s0089g00410	sucrose-phosphate synthase 1
VIT_18s0089g00420	unnamed protein product
VIT_18s0089g00450	PREDICTED: TMV resistance protein N-like isoform X1
VIT_18s0089g00460	unnamed protein product
VIT_18s0089g00490	unnamed protein product
VIT_18s0089g00500	unnamed protein product
VIT_18s0089g00510	unnamed protein product
VIT_18s0089g00520	PREDICTED: TMV resistance protein N-like isoform X1
VIT_18s0089g00530	unnamed protein product
VIT_18s0089g00540	unnamed protein product
VIT_18s0089g00550	PREDICTED: TMV resistance protein N-like
VIT_18s0089g00560	hypothetical protein VITISV_022891
VIT_18s0089g00570	unnamed protein product
VIT_18s0089g00590	hypothetical protein VITISV_022891
VIT_18s0089g00600	PREDICTED: TMV resistance protein N-like isoform X1
VIT_18s0089g00610	PREDICTED: uncharacterized protein LOC100248177
VIT_18s0089g00620	PREDICTED: glycerophosphodiester phosphodiesterase GDPD4 isoform X2
VIT_18s0089g00630	PREDICTED: receptor-like protein 12

VIT_18s0089g00650	PREDICTED: receptor-like protein 12
VIT_18s0089g00660	PREDICTED: LRR receptor-like serine/threonine-protein kinase GSO1
VIT_18s0089g00680	PREDICTED: receptor-like protein 12
VIT_18s0089g00690	PREDICTED: receptor-like protein 12
VIT_18s0089g00700	PREDICTED: cytochrome P450 714C2-like
VIT_18s0089g00710	hypothetical protein VITISV_039240
VIT_18s0089g00720	hypothetical protein VITISV_030083
VIT_18s0089g00730	hypothetical protein VITISV_030084
VIT_18s0089g00740	hypothetical protein VITISV_012392

Table S5. Primer sequences used in qRT-PCR analysis

**Gene ID**	**Forward Primers (5′-3′)**	**Reverse Primers (5′-3′)**
VIT_18s0041g00700	ATCGGGTGATTGGAGTGAAG	TGGCTGGTTTCTTCTCTCGT
VIT_18s0041g02140	GACCAAGCCAAAGAAGCACA	ATTGTTCCAGCCCCACCT
VIT_18s0041g02410	TGGGAACACGAAAGATGGAT	ATTGACAAGAGCCGAGAAGAGT
VIT_18s0089g00210	GCGGGTGACAATGTGAAGTT	TTGATGAGGTAGTCCGTGGC
Vvactin	CTTGCATCCCTCAGCACCTT	TCCTGTGGACAATGGATGGA
